# Osteoid Osteoma of the Great Toe Mimicking Osteomyelitis: A Case Report and Review of the Literature

**DOI:** 10.1155/2013/234048

**Published:** 2013-08-28

**Authors:** Ismail Turkmen, Bugra Alpan, Salih Soylemez, Feyza Unlu Ozkan, Koray Unay, Korhan Ozkan

**Affiliations:** ^1^Istanbul Medeniyet University Goztepe Training and Research Hospital, Department of Orthopaedics and Traumatology, 34732 Istanbul, Turkey; ^2^Istanbul Maslak Acibadem Hospital Department of Orthopaedics and Traumatology, 34000 Istanbul, Turkey; ^3^Istanbul Fatih Sultan Mehmet Training and Research Hospital, Department of Physcial Therapy and Rehabilitation, 34015 Istanbul, Turkey; ^4^Istanbul Medeniyet University, Department of Orthopaedics and Traumatology, 34732 Istanbul, Turkey

## Abstract

Osteoid osteomas are well-known benign tumors, seen generally in long bones. When seen in phalanxes or toes, they can cause a diagnostic dilemma. A young male presented to us with complaints of enlargement of the great toe and severe pain. He had had an ingrown toe-nail operation before, and this situation caused a diagnostic dilemma. In this case report, we emphasize that osteoid osteomas can cause diagnostic dilemmas and it should be kept in mind as a differential diagnosis.

## 1. Introduction

Osteoid osteoma is a small benign bone tumor, causing severe pain and pronounced sclerosis. It is seen most frequently in the proximal femur and the metaphyseal and diaphyseal parts of the tibia [1, 2]. An osteoid osteoma patient typically presents with throbbing night pain, which is dramatically relieved by salicylates. 

 Osteoid osteoma may be hard to differentiate clinically from subacute osteomyelitis and osteoblastoma. A subacute bone abscess may appear similar to osteoid osteoma in radiographs [3].

 An osteoid osteoma of the toe is quite rare [4]. In this paper, we present the case of a 23-year-old male patient who had previously undergone surgery for an ingrown toe nail and had undergone a second operation due to intractable pain, which was mostly assumed to be caused by osteomyelitis and yet was found to be related to osteoid osteoma in a pathological examination. Our aim in presenting this case is to emphasize that osteoid osteoma, however unusual, must be considered in the differential diagnosis of pathology mimicking osteomyelitis in the distal phalanx of the toe.

## 2. A Case Report

**Figure 1:**
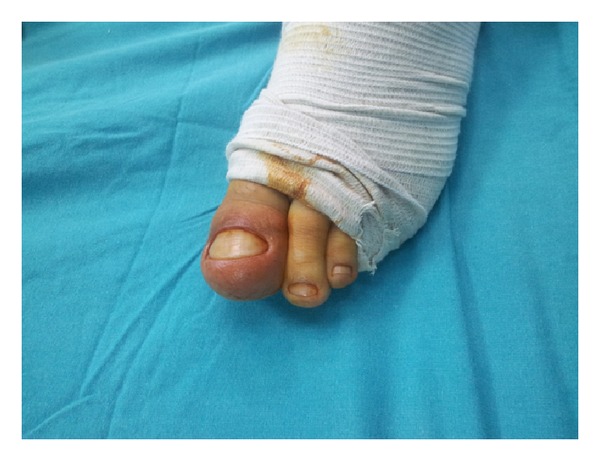
Preoperative clinical photograph of the left great toe.

A 23-year-old male patient presented to our outpatient clinic with complaints of ongoing pain of 1 year duration in the great toe of his left foot and not being able to wear a shoe due to the pain. His history revealed that he had undergone toe-nail extirpation by a general surgeon 2 years earlier and there was always pain when he did not take anti-inflammatories, especially at night. He subsequently used an unidentified antibiotic and oral etodolac 500 mg twice daily. In the physical examination, it was seen that his left great toe was enlarged when compared with the right side (Figure 1). The patient stated that his toe had been expanding for the last 2 years, while the pain started a year earlier. His toe was painful and warm on palpation. His past medical history and family history were unremarkable.

**Figure 2:**
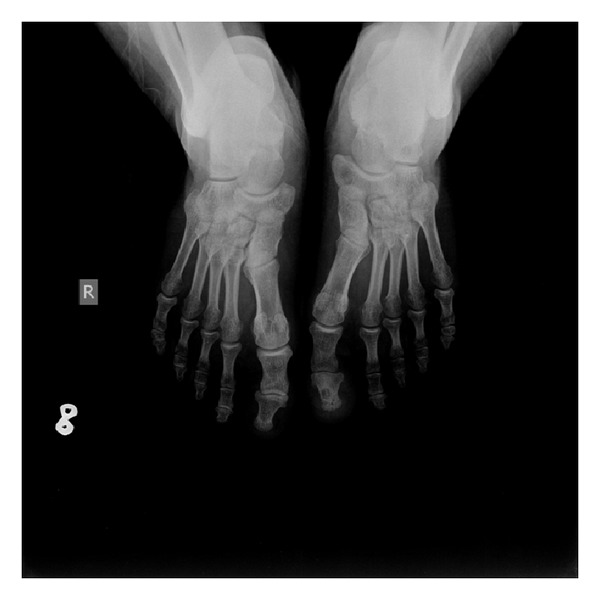
Preoperative AP X-ray of both feet.

**Figure 3:**
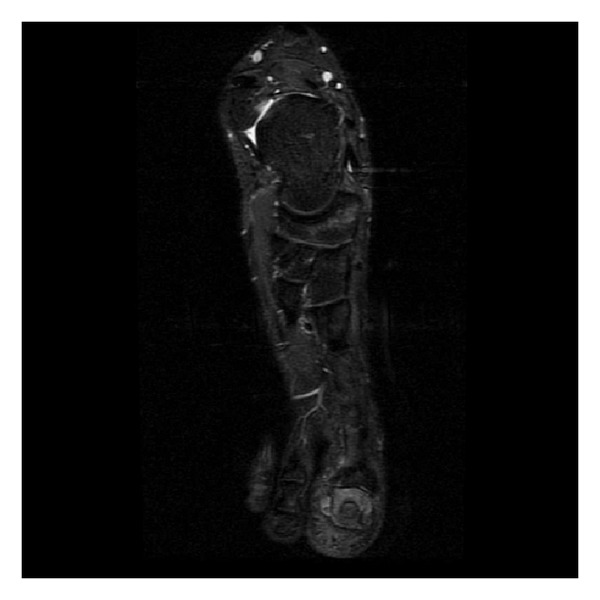
Preoperative coronal T1-weighted MR image of the left foot.

Preoperative erythrocyte sedimentation rate, C-reactive protein, and leukocyte values were all within normal ranges, while preoperative plain radiographs demonstrated a lytic bone lesion with a sclerotic rim in the medial part of the distal phalanx (Figure 2). Preoperative MRI showed bone marrow and soft tissue edema in the distal phalanx, compatible with osteomyelitis (Figure 3). ^99m^Tc scintigraphy showed increased activity in the left great toe.

**Figure 4:**
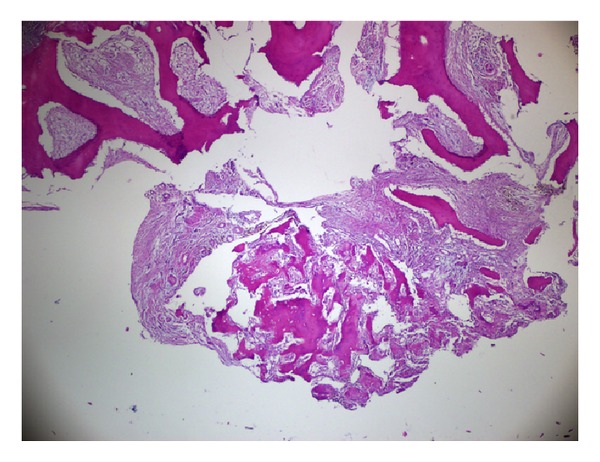
An osteoid osteoma nidus and the normal bone at the border of the lesion are demonstrated in the histological section. The nidus is composed of vascular fibrous tissue in which there are small, irregular, but connected, trabeculae of woven bone, demonstrating both prominent osteoblasts and prominent osteoclasts (H and E, ×4 obj.).

A longitudinal incision on the medial side of the distal phalanx was used for exposure. The tip of the distal phalanx was then excised with a rongeur. Soft and bony tissue samples were obtained for pathological examination and microbiological culture, with preoperative diagnoses of osteomyelitis and a neoplasm. Two musculoskeletal pathologists conducted histological examinations, revealing vascular fibrous tissue with spongious bone formation in the center, which was interpreted as the nidus of an osteoid osteoma (Figure 4). Microbiological cultures revealed no pathogenic organism. The patient's pain resolved immediately after the operation. Ten months have passed since the excision; the patient is well with no recurrence or symptoms.

## 3. Discussion

Osteoid osteoma is rarely seen in the toes [4–7]. Pain and swelling in the distal phalanx of the great toe may be attributed to a wide spectrum of diseases, such as benign and malignant bone neoplasms, cellulitis, and osteomyelitis. Osteomyelitis in the foot usually emerges as a complication of diabetes mellitus or due to progression of posttraumatic superficial infection [8]. Subacute osteomyelitis is characterized by insidious onset extremity pain, with no obvious finding of disease. Laboratory results are often not very helpful, and the radiological appearance may imitate benign or malignant tumors [9–11]. Osteomyelitis cases caused by recurrent ingrown toe nails have been reported [12, 13]. Osteomyelitis of the distal phalanx may be manifested by radiolucency of the bone and periosteal reaction. Pain and swelling of the great toe in the setting of a recurring ingrown toe nail led us to consider osteomyelitis first and a neoplastic event second.

Hypertrophy of the toes is a rare condition. Patients typically complain of pain, and their shoes becoming uncomfortable. The orthopedic literature describes etiologic factors causing enlargement of toes as intraosseous epidermoid inclusion cysts [14], pseudomonas osteomyelitis [15], subungual squamous cell carcinoma [16], macrodystrophia lipomatosa [17], chondrosarcoma [18], fibrolipoma [19], and osteoid osteoma.

Subungual squamous cell carcinoma is characterized radiologically by osteolysis and clinically by erythema and ipsilateral enlargement. In macrodystrophia lipomatosa, mesodermal tissues show hypertrophy, and the toe is observed to grow in both length and diameter. Fibrolipoma is a benign neoplasm that causes a soft tissue mass and usually does not cause radiologically apparent osseous changes. Chondrosarcoma in the toe phalanges may appear due to malignant transformation of an enchondroma. An epidermoid inclusion cyst is an expansile, radiolucent, and destructive bone lesion. In contrast, osteoid osteoma is a subcortical or intracortical bone lesion, with a diameter of up to 1 cm, and surrounded by a sclerotic rim [20]. Clubbing of the toe is usually observed on physical examination, and there is typically a history of night pain.

We reviewed English-language studies published between 1947 and 2012; 27 cases of osteoid osteoma in the phalanges of the toe were identified [4–6, 8, 21–39] (Table 1). Sherman [39] was the first to review the literature, finding six cases to which she added three of her own. However, that study did not report any data regarding the age or gender of the patients or which toes were involved. Our review of the literature revealed 13 osteoid osteoma patients with great toe involvement (four in the proximal and nine in the distal phalanges) [6, 21, 22, 24, 25, 28, 30, 32, 34, 35, 37, 38], seven patients with the second toe involved (two in the proximal and five in the distal phalanges) [5, 23, 27, 33, 36], four patients with the third toe involved (two in the proximal and two in the distal phalanges) [29, 39], two patients with the fourth toe involved (in the distal phalanges) [8, 26], and one patient with involvement of the proximal phalanx of the fifth toe [31]. Eighteen patients were males and nine were females. The mean age of the patients was 21.7 (range 9–38) years. The most common location was the great toe, while the fifth toe was the least frequent location. Four cases have been reported in the proximal phalanx of the great toe.

The most frequently involved phalanges were the distal ones, whereas involvement of the middle phalanges was not observed in any of the cases reported. Among the reported cases, there were twice as many male as female patients. The disease was observed most commonly in the second and third decades of life (Table 1).

Histopathological examination of osteoid osteomas demonstrates variably mineralized fine trabeculae of woven bone inside a central nidus. The caliber of trabeculae may vary. Benign osteoblastic border cells and multinucleated osteoclast-like giant cells are seen, dispersed in a fibrovascular stroma. Outside the nidus, fibrovascular tissue is surrounded by sclerotic lamellar bone.

Osteomyelitis is histopathologically distinguished from osteoid osteoma by the absence of a nidus and the presence of inflammatory cell infiltration. Osteoblastoma lacks a peripheral rim, and the lesion is usually >2 cm in diameter, whereas osteoid osteoma is usually <2 cm diameter. Osteosarcoma, on the other hand, lacks a fibrovascular stroma and an osteoblastic rim, while chondroid or fibrous differentiation may be present [40].

Our differential diagnoses were bone infections and benign bone lesions. Although we had performed an open excisional biopsy, considering the possibility of a neoplasm, a CT-guided biopsy might have been performed first.

Many hypotheses have been proposed for the mechanism of the intense pain in patients with osteoid osteoma. Wold et al. and Greco et al. found 500–1000 times higher concentrations of prostaglandin E2 (PGE2) in the nidus of osteoid osteomas compared with the surrounding normal bone [41, 42]. Also, toe hypertrophy may be explained by the increased level of PGE2, leading to increased expression of collagenase [43]. Mungo et al. also showed that both cyclooxygenase-1 (Cox-1) and cyclooxygenase-2 (Cox-2) enzymes are highly expressed in osteoblasts in osteoid osteoma, which are responsible for the high degrees of prostaglandin production [44]. 

Today, many lesions of the pelvis or long bones of the extremities can be treated with percutaneous radiofrequency ablation. The temperature at the tip of the electrode is increased to 90°C for 6 min. However, the procedure may not be indicated for lesions of the spine or lesions of the small bones of the hands and feet [45].

## 4. Conclusions

Osteoid osteoma is a benign tumor that is relatively easy to diagnose. However, it does not always present in a typical localization with characteristic clinical findings. This may cause difficulty in diagnosing this neoplasm. Osteoid osteoma must be kept in mind in the differential diagnosis of patients presenting with painful hypertrophy of the great toe. Furthermore, pathological sampling should never be ignored, even in patients for whom infection is considered the most likely diagnosis.

## Figures and Tables

**Table 1:** Review of the literature.

Case	Great toe	Second toe	Third toe	Fourth toe	Fifth toe	Total
Proximal phalanx	4	2	2	—	1	9
Middle phalanx	—	—	—	—	—	—
Distal phalanx	9	5	2	2	—	18
Age	9–30	12–20	17–25	38	38	Mean age: 21.7
Male	9	5	2	2	—	18
Female	4	2	2	—	1	9

Total no. of cases	13	7	4	2	1	27

## References

[1] McCarthy EF, Frassica FJ (1998). Primary bone tumors. *Pathology of Bone and Joint Disorders: with Clinical and Radiographic Correlation*.

[2] Frassica FJ, Waltrip RL, Sponseller PD, Ma LD, McCarthy EF (1996). Clinicopathologic features and treatment of osteoid osteoma and osteoblastoma in children and adolescents. *Orthopedic Clinics of North America*.

[3] McGrath BE, Bush CH, Nelson TE, Scarborough MT (1996). Evaluation of suspected osteoid osteoma. *Clinical Orthopaedics and Related Research*.

[4] Sproule JA, Khan F, Fogarty EE (2004). Osteoid osteoma: painful enlargement of the second toe. *Archives of Orthopaedic and Trauma Surgery*.

[5] Ebrahimzadeh MH, Omidi-Kashani F, Hoseini MR (2009). Painful and tender toe, osteoid osteoma of the distal phalanx of toe, a diagnostic dilemma. *Foot*.

[6] Onoue K, Kudawara I (2007). Osteoid osteoma with cartilage formation of the distal phalanx in the toe. *Orthopedics*.

[7] Tsang DSN, Wu D-Y (2008). Osteoid osteoma of phalangeal bone. *Journal of the Formosan Medical Association*.

[8] Laughlin RT, Reeve F, Wright DG, Mader JT, Calhoun JH (1997). Calcaneal osteomyelitis caused by nail puncture wounds. *Foot and Ankle International*.

[9] King DM, Mayo KM (1969). Subacute haematogenous osteomyelitis. *Journal of Bone and Joint Surgery B*.

[10] Lindenbaum S, Alexander H (1984). Infections simulating bone tumors: a review of subacute osteomyelitis. *Clinical Orthopaedics and Related Research*.

[11] Roberts JM, Drummond DS, Breed AL, Chesney J (1982). Subacute hematogenous osteomyelitis in children: a retrospective study. *Journal of Pediatric Orthopaedics*.

[12] Majcen ME, Wilfinger CC, Pilhatsch A (2009). Interpretation of radiologic abnormalities in patients with chronically infected ingrown toenails with regard to a possible exogenic osteomyelitis. *Journal of Pediatric Surgery*.

[13] Eisele SA (1994). Conditions of the toenails. *Orthopedic Clinics of North America*.

[14] Wang BY, Eisler J, Springfield D, Klein MJ (2003). Intraosseous epidermoid inclusion cyst in a great toe. A case report and review of the literature. *Archives of Pathology & Laboratory Medicine*.

[15] Brand RA, Black H (1974). Pseudomonas osteomyelitis following puncture wounds in children. *Journal of Bone and Joint Surgery A*.

[16] Nasca MR, Innocenzi D, Micali G (2004). Subungual squamous cell carcinoma of the toe: report on three cases. *Dermatologic Surgery*.

[17] Gupta SK, Sharma OP, Sharma SV, Sood B, Gupta S (1992). Macrodystrophia lipomatosa: radiographic observations. *British Journal of Radiology*.

[18] Koak YP, Patil PS, Mackenny RP (2000). Chondrosarcoma of the distal phalanx of a toe. A case report. *Acta Orthopaedica Belgica*.

[19] Milgram JW (1999). Massive fibrolipoma of a toe. *Journal of Foot and Ankle Surgery*.

[20] Ostrowski ML, Spjut HJ (1997). Lesions of the bones of the hands and feet. *American Journal of Surgical Pathology*.

[21] Hattori H, Takase K, Morohashi A (2011). Osteoid osteoma of the great toe. *Orthopedics*.

[22] Joett CR, Singh D (2010). Osteoid osteoma of the great toe: a case report. *Foot and Ankle Surgery*.

[23] Prietzel T, Hitzler P, Wojan M, Aigner T, von Salis-Soglio G (2009). Painful enlargement of the 2nd toe due to an osteoid osteoma in the distal phalanx. *Zeitschrift für Orthopädie und Unfallchirurgie*.

[24] Ozturk A, Yalçinkaya U, Ozkan Y, Yalçin N (2008). Subperiosteal osteoid osteoma in the hallux of a 9-year-old female. *Journal of Foot and Ankle Surgery*.

[25] Ekmekci P, Bostanci S, Erdoğan N, Akçaboy B, Gürgey E (2001). A painless subungual osteoid osteoma. *Dermatologic Surgery*.

[26] LaCroix ML, Thomas JR, Nicholas RW (2001). Subperiosteal osteoid osteoma of the distal phalanx of the fourth toe. *Orthopedics*.

[27] Lakkis S, Bazzi JS, Shabb NS (1998). Osteoid osteoma of the proximal phalanx of a toe: a case report. *Bulletin*.

[28] Barca F, Acciaro AL, Recchioni MD (1998). Osteoid osteoma of the phalanx: enlargement of the toe-two case reports. *Foot and Ankle International*.

[29] Wu KK (1991). Osteoid osteoma of the foot. *Journal of Foot Surgery*.

[30] Mohr VD, Bauer T, Schmitt B (1990). Osteoid osteoma of the terminal phalanx of the big toe. *Deutsche Medizinische Wochenschrift*.

[31] Shader AF, Schwartzenfeld SA (1989). Osteoid osteoma: report of a case. *Journal of Foot Surgery*.

[32] Alkalay I, Grunberg B, Daniel M (1987). Osteoid osteoma in an ossicle of the big toe. *Journal of Foot Surgery*.

[33] Le Saout J, Kerboul B, Courtois B (1984). Osteoid osteoma of the toes. Report on two cases. *Journal de Chirurgie*.

[34] Kahn MD, Tiano FJ, Lillie RC (1983). Osteoid osteoma of the great toe. *Journal of Foot Surgery*.

[35] Bordelon RL, Cracco A, Book MK (1975). Osteoid osteoma producing premature fusion of the epiphysis of the distal phalanx of the big toe. A case report. *Journal of Bone and Joint Surgery A*.

[36] Djian A, Valette F, Griffet G (1968). Osteoid osteoma of a toe. *Revue du Rhumatisme et des Maladies Osteo-Articulaires*.

[37] Reidy J, Hale CH, Sniffen RC (1945). Osteoid osteoma of terminal phalanx of right fifth toe. *The New England Journal of Medicine*.

[38] Hamilos DT, Cervetti RG (1987). Osteoid osteoma of the hallux. *Journal of Foot Surgery*.

[39] Sherman MS (1947). Review of the literature and report of thirty cases. *Journal of Bone and Joint Surgery*.

[41] Wold LE, Pritchard DJ, Bergert J, Wilson DM (1988). Prostaglandin synthesis by osteoid osteoma and osteoblastoma. *Modern Pathology*.

[42] Greco F, Tamburrelli F, Ciabattoni G (1991). Prostaglandins in osteoid osteoma. *International Orthopaedics*.

[43] Renò F, Grazianetti P, Cannas M (2001). Effects of mechanical compression on hypertrophic scars: prostaglandin E2 release. *Burns*.

[44] Mungo DV, Zhang X, O’Keefe RJ, Rosier RN, Edward Puzas J, Schwarz EM (2002). COX-1 and COX-2 expression in osteoid osteomas. *Journal of Orthopaedic Research*.

[45] Lindner NJ, Ozaki T, Roedl R, Gosheger G, Winkelmann W, Wörtler K (2001). Percutaneous radiofrequency ablation in osteoid osteoma. *Journal of Bone and Joint Surgery B*.

